# Optimization pilot scale study on ammonia nitrogen removal by bio filter

**DOI:** 10.1038/s41598-023-42885-6

**Published:** 2023-09-20

**Authors:** Junlong Meng, Songtao Shen, Chuanhui Zhou, Tuodi Zhang, Yingyi Xu

**Affiliations:** 1 Present Address: Southwest University of Science and Technology, Mianyang, 621010 China; 2 Tianfu College of Southwestern University of Finance and Economics, Mianyang, 621000 China

**Keywords:** Ecology, Environmental sciences, Engineering

## Abstract

In order to obtain the optimal conditions for ammonia nitrogen (AN) wastewater treatment by bio filter (BF), the effects of ratio of carbon to nitrogen (C/N), pH, and hydraulic load (HL) on the AN degradation were studied by Response Surface Methodology (RSM). Central Composite Design (CCD) experiments were conducted, and the response of the AN removal rates were fitted to a second-order polynomial model. The analysis of variance showed that the model was accurate and reliable. Through model fitting, the optimal condition for AN removal was: C/N of 18.95, pH of 7.78, and HL of 1.04 d^−1^. The maximum AN removal rate predicted by the model was 91.90%, accorded with the experimental verification value of 91.37% under the optimal condition. The research provided valuable demonstration for optimizing process parameters on AN removal in BF.

## Introduction

On the premise that centralized sewage treatment plants have been built and operated in urban areas of the country, rural wastewater has become an important source of water environment pollution, and has also become a problem that hinders the continuous improvement of water environment quality in China, especially in the relatively backward western regions^1^. The rural wastewater comes from many aspects, such as pesticides and fertilizers, rural domestic sewage, husbandry and aquaculture, biogas fermentative liquid etc. It has the characteristics of decentralized pollution sources, difficult collection, small treatment scale and large changes in water quantity and quality^2^. It is impossible to build large-scale centralized wastewater treatment plants, and decentralized treatment is the main choice^3^. Bio filter (BF) is a typical ecological treatment technology, widely used in rural areas of China for wastewater treatment due to its advantages of simple structure, low energy consumption, easy operation and maintenance, and strong impact load resistance^4^. However, many rural wastewater treatment facilities applied bio filter technology cannot make a satisfied result, especially for ammonia nitrogen (AN) removal^5^.

**Figure 1:**
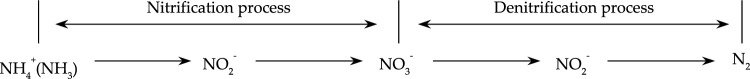
The basic principle of AN removal.

AN in wastewater is a nitrogen compound that exists in the form of ammonia (NH_3_) or ammonium ions (NH_4_^+^). It can cause eutrophication, oxygen depletion, undesirable odor and other problems in water bodies. The effective removal of AN is beneficial for the health and equilibrium of ecosystem of water. The basic principle of AN removal (Fig. 1) in BFs is to use granular fillers and biofilms attached to the surface of the fillers as the main treatment medium. When wastewater flows through, the fillers and biofilm will adsorb NH_3_ and NH_4_^+^ on its surface, and complete nitrification and denitrification under the action of nitrifying bacteria and denitrifying bacteria, so as to achieve the effect of AN removal^6^.

Domestic and foreign scholars^7,8^ have conducted extensive research on the influencing factors of AN removal in BFs, such as plants^9^, filter media^10^, carbon releasing materials^11^ etc. For the BF reactor that has been constructed, hydraulic load (HL), ratio of carbon to nitrogen (C/N) and pH three factors received the widespread attention. Ren et al.^12^ pointed out that when the pH was kept within 7–8, the denitrification process was well, and pH > 8 or pH < 7 would affect the activity of denitrification bacteria, thereby affecting the denitrification process. Nagaoka et al.^13^ suggested that when the C/N in wastewater was more than or equal to 5.0, the carbon source required for denitrification process can be ensured. When the C/N was less than 5.0, the external carbon source dosage should be increased. In the research on the treatment of urban wastewater by the BF, Dou^14^ found that the removal rate of NH_3_-N gradually increased, as the HL varied from 2.3 to 3.3 m^3^/(m^2^ h); when the HL changed from 3.3 to 3.9 m^3^/(m^2^ h), the removal rate of NH_3_-N gradually decreased from 86 to 73%. There are many researches on the influencing factors of AN removal, but more researches concentrated on the independent analysis of single factor, rarely involving effects of multiple factors and their interactions. Furthermore, in order to achieve better AN removal rate, the optimized comprehensive conditions of pH, C/N and HL were also worthy of further investigation.

In traditional single factor optimization, a single variable was tested while holding all other influencing factors constant. However, this method became tedious and laborious when dealing with multivariate systems, sometimes even leading to incorrect conclusions^15^. Response Surface Methodology (RSM) is a suitable tool for solving nonlinear data processing problems^16^. It includes various techniques such as design of experiments, modeling, suitability evaluation, and determination of the optimal combination conditions. By performing regression fitting of the process and plotting the response surface and contour lines, it becomes easy to obtain the response values corresponding to each factor level. This information can then be used to identify the optimal predicted response value and the corresponding experimental conditions^17^. This means that RSM can provide sufficient information about the impact of the factors by performing fewer experiments^18^. In this study, we focused on the removal of AN in a BF, and RSM was used to optimize three factors, namely HL, pH, and C/N of the influent wastewater to maximize the removal rate of AN in the BF, in the hope of providing the references for rural wastewater treatment.

## Materials and methods

### Experimental equipment and materials

**Figure 2:**
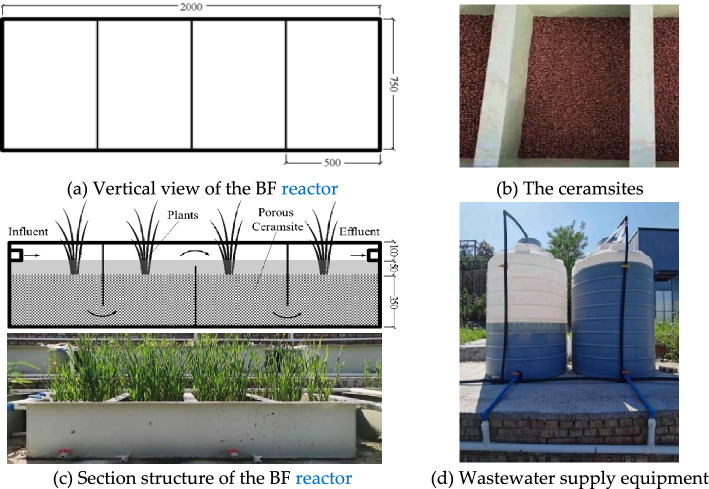
Pilot scale experimental equipment in this research.

The BF reactor (pilot scale experimental equipment in this research) was shown in Fig. 2.

The main treatment device was made of polypropylene, rectangular parallelepiped shaped, with the size of 2000 mm × 750 mm × 500 mm. The porous ceramic particles, 8 mm in diameter, were packed into the device with a filling depth of 350 mm. The porous ceramic particles on which grow the calamus were submerged 50 mm below the water level. The treatment device was also equipped with two sets of wastewater supply equipment, one work and one backup. Each set of equipment was composed of a plastic bucket with a capacity of 1000 L and a flow rate controller.

In this experiment, the AN wastewater was prepared with ammonium chloride which was purchased from Jinshan Chemical Reagent (Chengdu, China). Glucose and sodium carbonate as the carbon resource and pH regulator respectively, were purchased from Kelong Chemical Reagent (Chengdu, China). All other chemicals (e.g., sulfuric acid, hydrochloric acid, phosphoric acid, sodium hydroxide, potassium persulfate, mercury iodide) were purchased from Xinjie Chemical Reagent (Mianyang, China).

### Experimental procedure and analysis methods

In order to eliminate the influence of other factors as much as possible, all experiments were scheduled to be conducted on rainless days in May 2023. After the BF reactor had been running stably, the effluent sample with the temperature of 20 ± 0.5 °C was collected, usually around mid-afternoon. AN was detected by Nessler’s reagent spectrophotometry, and its removal rate was calculated using the equation as follows.

1 $$ \eta = \frac{{C_{i} - C_{e} }}{{C_{i} }} \times {100} $$

where η was the removal rate of AN. C_i_ and C_e_ represented the AN concentration of influent and treated effluent respectively.

### Experimental design and optimization method

Central Composite Design (CCD) is a commonly used design in RSM, especially suitable for sequential experiments. CCD is an ideal solution to fit a second order response surface that can be used to study the effect of multiple factors at different levels. The rotation and sequentiality of the experiments can be ensured by using the proper axial points in CCD^19^. CCD is designed with five levels, with level codes of 0, ± 1, and ± α, where 0 is the median, α is an extreme value. For the three experimental factors (C/N, pH and HL), α is $2^{3/4}$, 1.68. Experimental levels and factors of CCD were presented in the Table 1.

**Table 1:** Experimental levels and factors of CCD.

Coded level	Uncoded level		
	C/N	pH	HL (d^−1^)
− 1.68	11.59	6.66	0.16
− 1	15.00	7.00	0.50
0	20.00	7.50	1.00
1	25.00	8.00	1.50
1.68	28.41	8.34	1.84

For a CCD involving three factors with three levels each, a total of 20 sets of experiments were necessary. These experimental sets consisted of 8 star point experiments, 6 axis point experiments, and 6 center point experiments. The CCD experimental data were analyzed and the response of the AN removal rate was fitted to a universal model, incorporating three experimental factors. The second-order polynomial model was depicted in Eq. (2).

2 $$ \eta = C_{0} + \sum\limits_{i = 1}^{n} {C_{i} X_{i} } + \sum\limits_{i = 1}^{n} {C_{ii} X_{i}^{2} } + \sum\limits_{1 \le i < j \le n}^{n} {C_{ij} X_{i} X_{j} } $$

where η represented the removal rate of AN, the dependent variable, and *X* was the independent variable (C/N, pH or HL). C_*0*_ was the constant term, and the linear coefficient was represented by *C*_*i*_. *C*_*ii*_ and *C*_*ij*_ represented the square term coefficient and the interaction coefficient respectively.

## Results and discussion

### Model fitting

The CCD experiment was divided into 20 experimental runs, and each run conducted three parallel tests to calculate the average removal rate of AN (see Table 2). All the removal rates of AN were between 51.35 and 92.09%, and the average value was 74.48%. In center runs of the CCD experiments, AN removal rates varied from 87.05 to 92.09% with the average value of 89.26%, much higher than the ones calculated from other runs (Star and Axial), with the average value of 68.14%. The rotation and sequentiality of the experiments were well demonstrated.

**Table 2:** The CCD experimental conditions and corresponding AN removal rates.

Run	Type	Uncoded Level			AN removal rate (%)
		C/N X_1_	pH X_2_	HL (d^−1^) X_3_	
1	Star	15	7	0.5	65.30
2	Star	25	7	0.5	59.45
3	Star	15	8	0.5	80.35
4	Star	25	8	0.5	69.21
5	Star	15	7	1.5	57.35
6	Star	25	7	1.5	58.58
7	Star	15	8	1.5	76.89
8	Star	25	8	1.5	76.08
9	Axial	11.59	7.5	1	73.15
10	Axial	28.41	7.5	1	56.84
11	Axial	20	6.66	1	51.35
12	Axial	20	8.34	1	82.15
13	Axial	20	7.5	0.16	69.97
14	Axial	20	7.5	1.84	77.33
15	Center	20	7.5	1	87.05
16	Center	20	7.5	1	89.83
17	Center	20	7.5	1	89.50
18	Center	20	7.5	1	92.09
19	Center	20	7.5	1	88.28
20	Center	20	7.5	1	88.78

*AN* ammonia nitrogen.

With the assistance of Design-Expert 10.0.8.0, the predictive quadratic polynomial regression model was developed, and its coefficients were figured out with least square method. The unencoded numerical value was assigned to including constant term, the linear coefficient, the square term coefficient and the interaction coefficient respectively, forming the following prediction equation.

3 $$ \begin{aligned} \eta & =  - 1929.01 \, + \, 14.72X_{1} + \, 486.45X_{2} - \, 19.29X_{3} \hfill \\ & \quad - \, 0.37X_{1} \cdot X_{2} + \, 0.87X_{1} \cdot X_{3} + \, 6.11X_{2} \cdot X_{3} \hfill \\ & \quad - \, 0.34X_{1}{^{2}} - \, 31.24X_{2}{^{2}} -  21.48X_{3}{^{2}} \hfill \\ \end{aligned} $$

In this equation, *η* was the AN removal rate and* X*_2_ stood for pH. *X*_1_ and *X*_3_ represented the C/N and HL of the influent.

Through variance analysis, the predictive quadratic polynomial regression model of the AN removal rate (Eq. 3) was evaluated the quality of the fitting degree, and the results were listed in the Table 3. The F-value is the ratio of inter group mean square to intra group mean square in analysis of variance. At a certain level of confidence, it can be used to evaluate whether the difference between two sets of data comes from systematic differences or random errors. The F-value and p-value can reflect the significant impact of each control factor in the model, and the larger the F-value and the smaller the p-value, the more significant the correlation^20^. The F-value of the model was 45.00 and the p-value less than 0.0001, which showed that the model was highly significant and fitted well throughout the studied regression region. Meanwhile, the P-value of the mismatch term was 0.067, and the mismatch of the model is not significant. The R^2^ (goodness of fit) was used to test the density of sample data points clustered around the regression line, and to evaluate the fitting degree of the regression model to the sample observation values. The R^2^ was 0.976, indicating that the model could explain the change in response value of 97.6%, and the variation in removal rate of less than 3% could not be explained by the model. The coefficient of variation, also known as the standard deviation, is the ratio of the standard deviation to the mean, abbreviated as CV. The CV was 3.72% less than 10%, which indicated that the results of CCD experiments were reliable^21^. The Adeq precision was 18.4, much higher than 4.0, which implied that the CCD experimental results were precise^22^. From the influence of variables simulated by the regression model, The effect of *X*_2_ (pH) on the AN removal rate was linearly significant (p < 0.05). However, in the pairwise interaction of three variables (C/N, pH, and HL), HL-C/N effect was significant (p < 0.05), others were not significant.

**Table 3:** The variance analysis of the fitting model.

Source	Sum of squares	DF	Mean square	F value	P value
Model	3110.26	9	345.58	45.00	< 0.0001
X_1_	141.90	1	141.90	18.48	0.0016
X_2_	945.73	1	945.73	123.15	< 0.0001
X_3_	3.56	1	3.56	0.46	0.5113
X_1_·X_2_	6.72	1	6.72	0.87	0.3717
X_1_·X_3_	37.95	1	37.95	4.94	0.0404
X_2_·X_3_	18.69	1	18.69	2.43	0.1498
X_1_^2^	1024.40	1	1024.40	133.40	< 0.0001
X_2_^2^	879.00	1	879.00	114.46	< 0.0001
X_3_^2^	415.75	1	415.75	54.14	< 0.0001
Residual	76.79	10	7.68		
Lack of fit	62.37	5	12.47	4.32	0.0670
Pure error	14.43	5	2.89		
Cor total	3187.05	19			

Residual is the difference between the experiment result and the regression model fitting. In the regression analysis, the residuals conforming to the normal distribution is pre assumed. Therefore, conducting a normality test of residuals can ensure that the assumptions of regression analysis are valid, thereby ensuring the accuracy of model fitting. Residual plots can reveal experimental data structures and discover information that numerical results cannot provide, making them an indispensable part of model diagnosis^23^.

**Figure 3:**
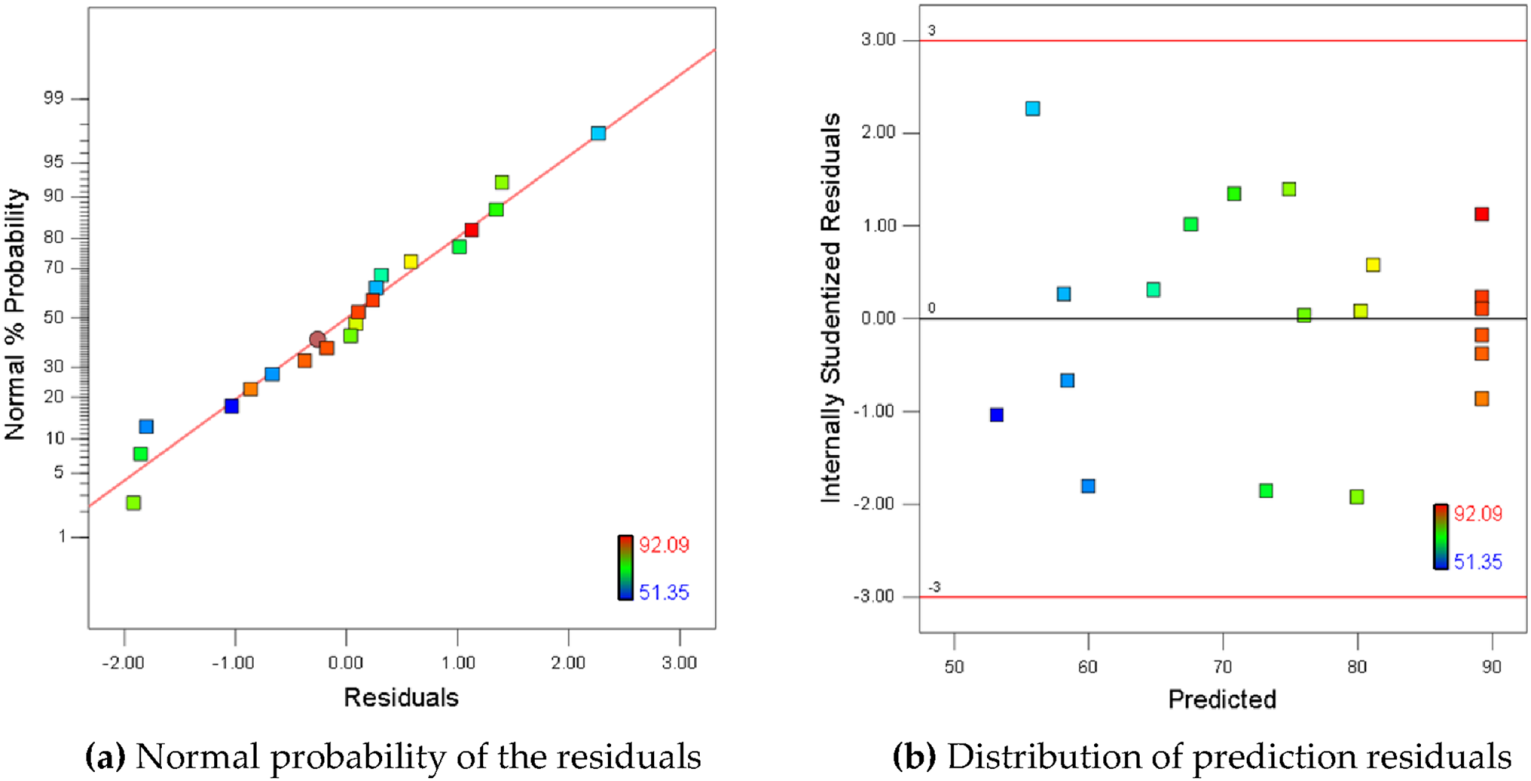
The distribution of residuals.

**Figure 4:**
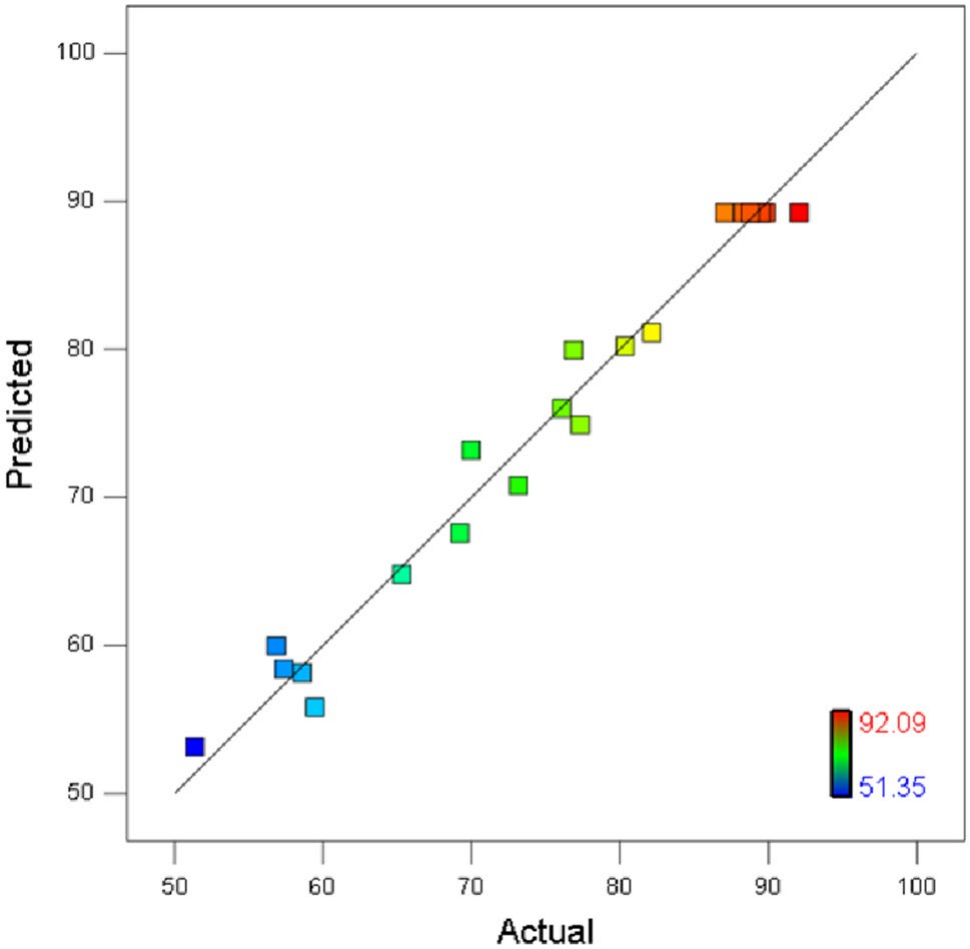
The actual and predicted values of AN removal rates.

Figure 3a showed the relationship between the cumulative frequency distribution of the standardized residuals and the cumulative probability distribution of Normal distribution. The points in the figure were distributed in an approximate straight line, indicating that the normal distribution assumption was acceptable. The distribution of residuals with predicted values exhibited significant randomness (Fig. 3b), and there was no correlation between adjacent residuals. These implied that the residuals did not contain any predictable information^24^. From the relationship between actual and predicted values (Fig. 4), the data points distributed along the predicted diagonal. This suggested that the actual AN removal rates were highly consistent with the predicted values of the regression model.

**Figure 5:**
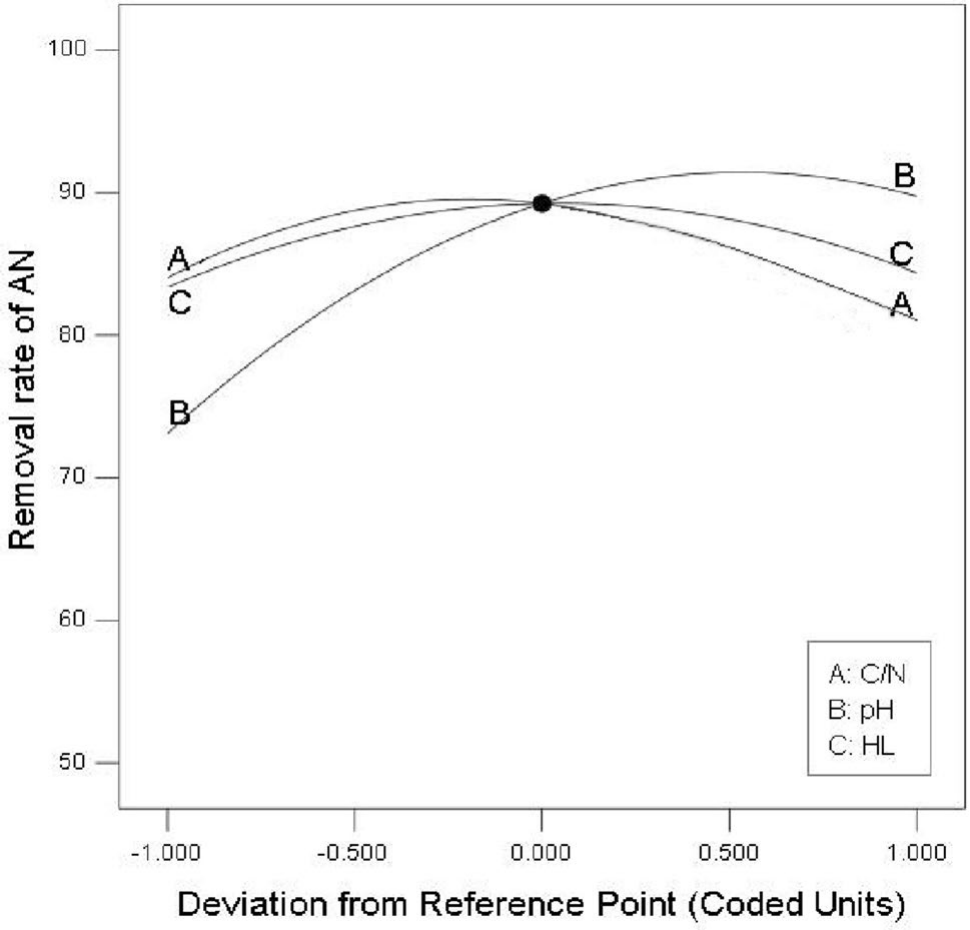
The influence of three factors on the AN removal rate.

In order to compare the influence of three factors on the AN removal rate at the same scale, three curves (Fig. 5) were obtained in which, horizontal-axis was for coded variation of C/N, pH and HL, vertical-axis was for the AN removal rate. The variation characteristics of the three curves were similar, all rising first and then decreasing, showing the single-peaked trends. As each of the three factors changed in the CCD experimental range, the AN removal rate can reach the corresponding maximum value. The synergistic changes between the C/N curve and the HL curve were evident, both reaching their peaks near the CCD center point with coded level of 0. This indicated a significant interaction between C/N and HL, consistent with the results of the previous analysis of variance.

### Influence factor of AN removal rate

**Figure 6:**
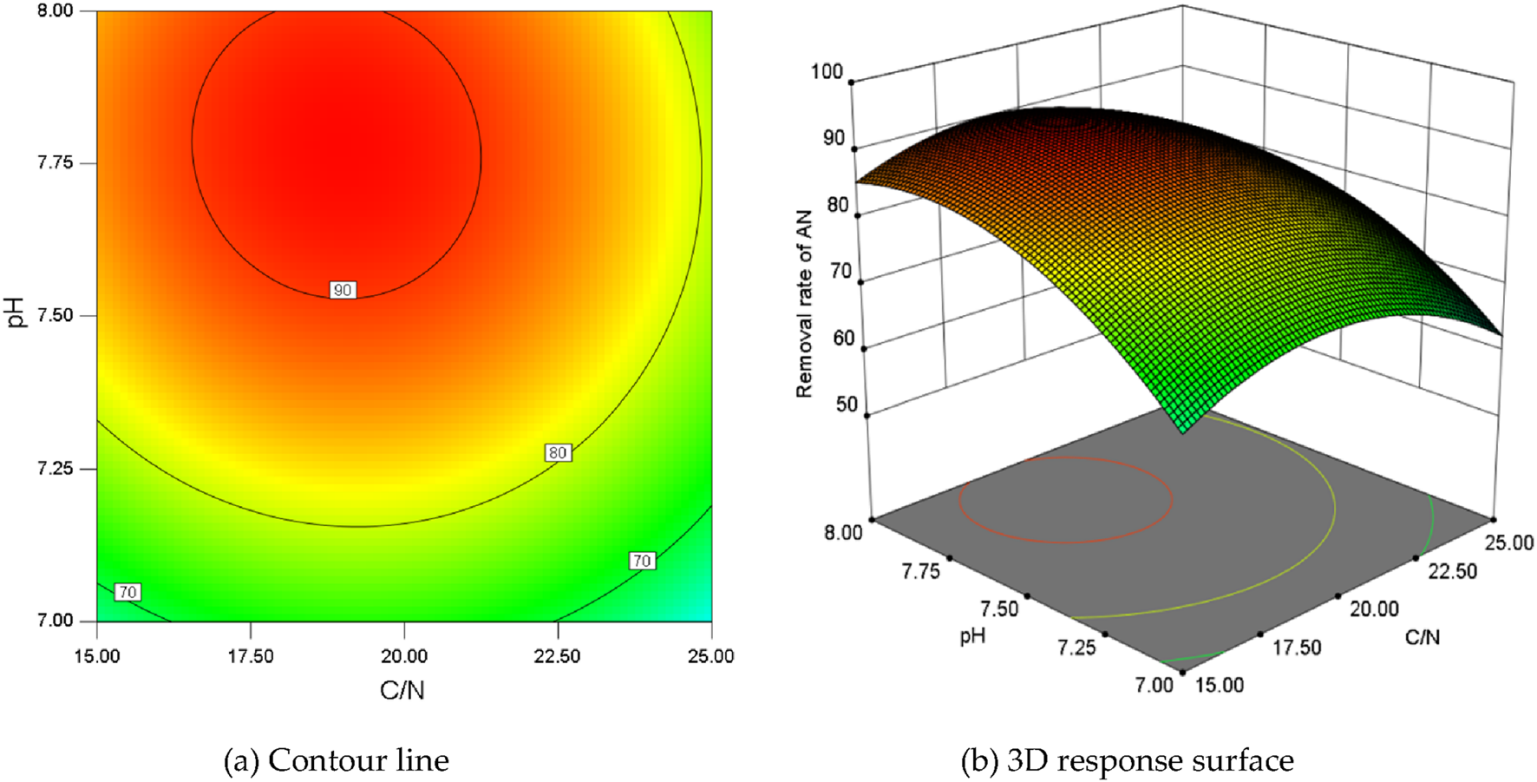
The interaction effect between pH and C/N at HL = 1 d^−1^.

The contour line diagram and 3D response surface diagram could intuitively reflect the influence of the interaction of various factors on the response value^25^. Figure 6 showed the interaction between pH and C/N on the AN removal rate under the central value condition of HL. Within the same coded unit range, the removal rate variation range of the vertical-axis was larger than that of the horizontal-axis (Fig. 6a), suggesting that pH had a greater impact on the AN removal rate than C/N. The curvature of the pH direction surface was higher than that of the C/N direction (Fig. 6b), and the effect of pH on AN removal rate was dominant, with no significant interaction between the two.

**Figure 7:**
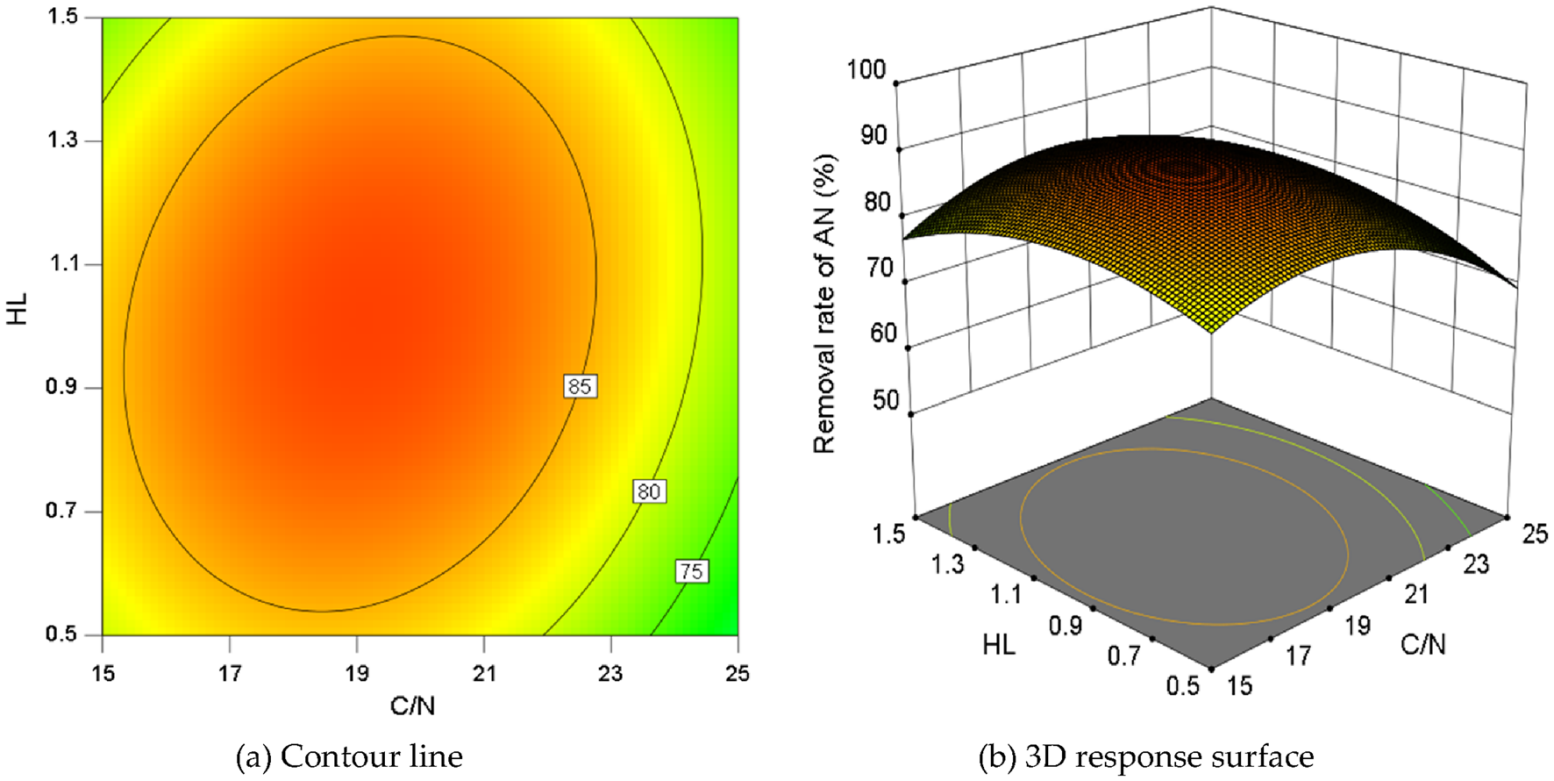
The interaction effect between HL and C/N at pH = 7.50.

Different situation occurred in the interaction between HL and C/N on the AN removal rate under the central value condition of HL (Fig. 7). Within the same coded unit range, the change range of removal rate on the vertical-axis was close to that on the horizontal-axis (Fig. 7a), implying that the effect of HL on AN removal rate was similar to that of C/N. The curvature of the surface differed slightly in the HL and C/N directions (Fig. 7b), and the synergistic effect on the AN removal rate appeared between HL and C/N., with significant interaction.

**Figure 8:**
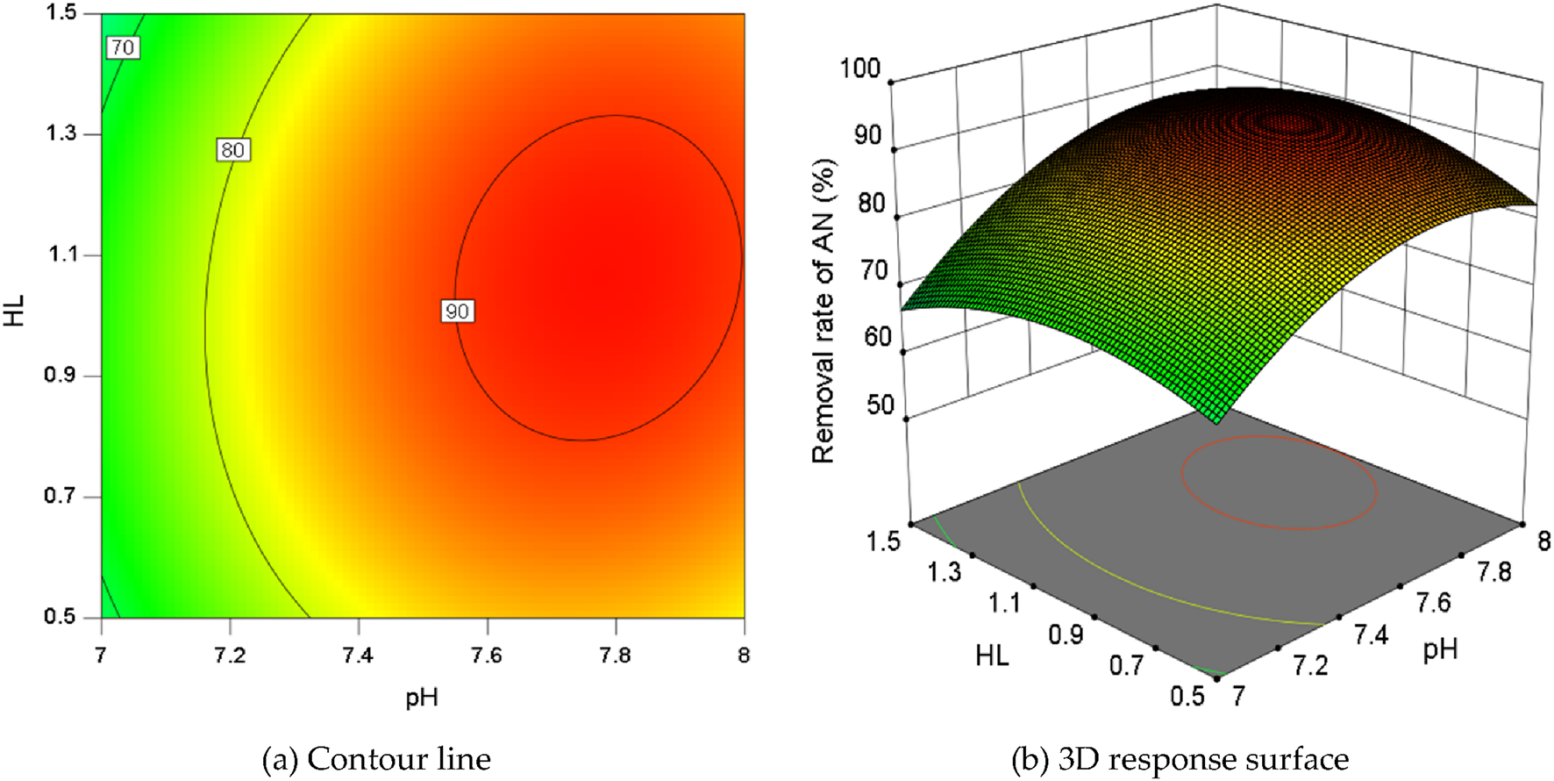
The interaction effect between HL and pH at C/N = 20.

Within the same coded unit range, the removal rate variation range of the vertical-axis was less than that of the horizontal-axis (Fig. 8a), indicating that pH had a greater impact on the AN removal rate than HL. The curvature of the pH direction surface was higher than that of the C/N direction (Fig. 8b), and the effect of pH on AN removal rate was dominant, with no significant interaction between the two.

The optimal condition for AN removal (Table 4) was obtained through model fitting: C/N of 18.95, pH of 7.78, and HL of 1.04 d^−1^. To verify the accuracy of the prediction model, three parallel validation experiments were conducted under optimal condition in the BF, and the average AN removal rate was calculated to be 91.37%, which was not significantly different from the predicted value (91.90%) of the model.

**Table 4:** The optimal solution and validation of the optimization model.

Optimum conditions			AN removal rate (%)	
C/N	pH	HL (d^−1^)	Experimental	Predicted
18.95	7.78	1.04	91.37	91.90

The pH affected nitrification and denitrification processes greatly. Pramanik^26^ found that for the nitrification process, the removal rate of NH_3_-N ranged from 61.32 to 90.24% when the pH was 6–9; When the pH of the influent was between 7.2 and 8.6, the removal rate of NH_3_-N reached the highest, basically maintaining around 90%; When pH < 7.2 or pH > 8.6, the removal rate of NH_3_-N decreased, and the nitrification process was inhibited. Carbon source is the largest nutrient source for microorganisms, and the C/N has a significant impact on the activity of both nitrifying and denitrifying bacteria. Through the experiment, Guo^27^ investigated the effects of C/N on simultaneous nitrification and denitrification in BF. The results indicated that when the C/N was too low, the denitrification carbon source was insufficient; when the C/N is too high, a large number of carbonized heterotrophic bacteria multiply, inhibiting the growth of nitrifying bacteria, leading to a decrease in nitrification efficiency; these all could reduce the effect of nitrogen removal. Different from the first two factors, HL had a significant impact on the balance between nitrification and denitrification processes. Using actual domestic sewage as the study object, Wei et al.^28^ studied the impact of HL on treatment efficiency of BF. The experimental results indicated that with the increasing of HL, the removal rate of NH_3_-N first increased then decreased. The above studies unilaterally confirm the results of this research in terms of their impact on the AN removal. For the BF reactor that has been constructed, HL, C/N and pH three engineering parameters should be comprehensively considered, and the interaction between them could not be ignored. In this way, the optimized parameters can effectively improve the removal rate of AN.

## Conclusions

In order to obtain the optimal conditions for the AN wastewater treatment by the self-built BF, the factors influencing the removal of AN were studied by using RSM. The main conclusions are as follows: (1) Using C/N, pH, and HL as influencing factors and AN removal rate as response values, a regression model was established. The model was significant, and the mismatch term was not significant. The model was accurate and reliable, with less than 3% variation that could not be explained by this model. (2) Through model fitting, the optimal condition for AN removal was: C/N of 18.95, pH of 7.78, and HL of 1.04 d^−1^. After experimental verification in the BF, the average AN removal rate (91.37%) accorded with the model prediction value (91.90%) under the optimal condition. The research provided valuable demonstration for optimization of AN degradation process parameters in wastewater treatment.

## Data Availability

The data is included in this article. For more information, please contact Songtao Shen (shenpaul@126.com).
